# Consequences of mutation accumulation for growth performance are more likely to be resource-dependent at higher temperatures

**DOI:** 10.1186/s12862-021-01846-1

**Published:** 2021-06-06

**Authors:** Xiao-Lin Chu, Quan-Guo Zhang

**Affiliations:** State Key Laboratory of Earth Surface Processes and Resource Ecology and MOE Key Laboratory for Biodiversity Science and Ecological Engineering, College of Life Sciences, Beijing Normal University, Beijing, 100875 People’s Republic of China

**Keywords:** Mutational effects, Genotype-by-environment interaction, Conditional neutrality, Deleterious mutation, Neutral mutation, Local adaptation

## Abstract

**Background:**

Mutation accumulation (MA) has profound ecological and evolutionary consequences. One example is that accumulation of conditionally neutral mutations leads to fitness trade-offs among heterogenous habitats which cause population divergence. Here we suggest that temperature, which controls the rates of all biochemical and biophysical processes, should play a crucial role for determining mutational effects. Particularly, warmer temperatures may mitigate the effects of some, not all, deleterious mutations and cause stronger environmental dependence in MA effects.

**Results:**

We experimentally tested the above hypothesis by measuring the growth performance of ten *Escherichia coli* genotypes on six carbon resources across ten temperatures, where the ten genotypes were derived from a single ancestral strain and accumulated spontaneous mutations. We analyzed resource dependence of MA consequences for growth yields. The MA genotypes typically showed reduced growth yields relative to the ancestral type; and the magnitude of reduction was smaller at intermediate temperatures. Stronger resource dependence in MA consequences for growth performance was observed at higher temperatures. Specifically, the MA genotypes were more likely to show impaired growth performance on all the six carbon resources when grown at lower temperatures; but suffered growth performance loss only on some, not all the six, carbon substrates at higher temperatures.

**Conclusions:**

Higher temperatures increase the chance that MA causes conditionally neutral fitness effects while MA is more likely to cause fitness loss regardless of available resources at lower temperatures. This finding has implications for understanding how geographic patterns in population divergence may emerge, and how conservation practices, particularly protection of diverse microhabitats, may mitigate the impacts of global warming.

**Supplementary Information:**

The online version contains supplementary material available at 10.1186/s12862-021-01846-1.

## Background

Deleterious and neutral (or nearly neutral) mutations can get fixation by drift in populations; and mutation accumulation (MA) has important ecological and evolutionary consequences [1, 2]. Accumulation of deleterious mutations increases the magnitude of maladaptation and thus the chance of population extinction [3, 4]. On the other hand, MA can increases cryptic genetic diversity which may fuel adaptation to changing environments or break the constraints on future adaptive mutations [5–8]. Crucially, fitness effects of mutations could be environment-dependent [9–12], and accumulation of conditionally neutral mutations (that show neutral fitness effects in one specific environment but are deleterious in alternate environments) plays an important role for the emergence of local adaptation, and thus population divergence, across habitats [13–18].

The environmental dependence of MA effects has been investigated by a large body of research; and most studies find that stressful environmental conditions typically magnified mutational effects (increasing the variance) [19–23]. While the comparisons between benign and obviously stressful environments (involving factors such as antibiotics, temperature, osmolarity and pH) are clear-cut, it remains poorly understood how prevalent environmental dependence in MA effects is on a finer scale of environmental heterogeneity. This question is crucial for understanding the evolution of fitness trade-offs among natural populations which are often located in relatively benign environments with only subtle differences, e.g., in substitutable resources [24, 25].

Here we suggest that temperature, which controls the rates of all biochemical and biophysical processes, should play a crucial role for determining mutational effects, and particularly the environment-dependence of MA effects. As described by the Arrhenius equation [26], higher temperatures universally speed the rate-limiting processes such as resource uptake, DNA replication and protein synthesis [27–30]. Consequently, higher temperatures may mitigate the effects of some, albeit not all, deleterious mutations, e.g., those involved in nutrient acquisition [31, 32]. Taking resource use as an example, we may have a specific prediction that MA should cause reduced capacities of using multiple resources at lower temperatures, and impact the utilization of fewer resources at relative higher temperatures. Therefore, higher temperatures should increase the chance of conditional neutrality, or more generally speaking, genotype-by-environment interaction, in MA effects (see Additional file 1: Fig. S1 for a graphical illustration of this hypothesis by drawing fitness landscapes). Note that our hypothesis is based on temperature effects on rate-limiting processes; and therefore it may hold for’normal’ temperature ranges that natural populations are typically faced with [33, 34], but not stressfully high temperatures where protein thermal stability, instead of the rates of physiological processes, may determine growth performance [35–37].

## Results

**Fig. 1:**
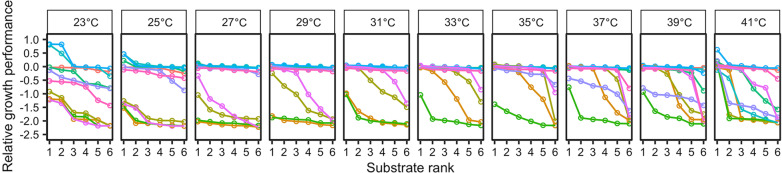
Rank of carbon substrates by relative growth performance at each temperature. Each line shows the relative growth of a single genotype across different substrates, with substrates ranked by decreasing relative growth scores of each individual genotype. Note that the substrates at a given rank may be different for different genotypes. Increased separation between lines indicates larger genetic variance and greater steepness of the lines implies increased environmental variance

**Fig. 2:**
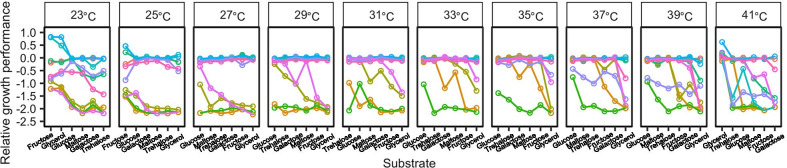
Rank curves of carbon substrates with fixed orders to show responsiveness and inconsistency in genotype-by-environment variance. Each line represents the relative growth of a genotype across different substrates. The order of substrates at each temperature is ranked by decreasing growth performance of the genotype with the highest mean growth values across all substrates. The discrepancy in slope among genotypes represents responsiveness (unequal variances on different substrates); and intersection of the lines represents inconsistency

The present study examined how temperature alters MA consequences for resource use in *Escherichia coli.* We estimated the abilities of bacterial MA genotypes to use six carbon substrates by measuring growth yields, as earlier studies [24, 38, 39]. The measurement was carried out at ten temperatures. Relative growth performance scores of the MA genotypes were typically negative, suggesting that MA genotypes generally showed reduced, not increased, growth yields compared with the ancestral type (Figs. 1, 2; Additional file 1: Table S1; note that no relationship was found between the number of base-pair substitutions and growth performance; Additional file 1: Tables S2 and S3). Relative growth performance differed among genotypes (Figs. 1, 2; linear mixed-effect model, *χ*^*2*^_9,522_ = 1213.076, *P* < 2 × 10^–16^), and carbon resources (*χ*^*2*^_5,522_ = 27.880, *P* = 4 × 10^–5^). Temperature showed a positive linear effect (*χ*^*2*^_1,522_ = 22.119, *P* = 3 × 10^–6^) and a negative quadratic effect (*χ*^*2*^_1,522_ = 20.620, *P* = 6 × 10^–6^); this suggests that intermediate temperatures (~ 25 to ~ 35 °C) allowed MA genotypes to show greater relative growth performance (that is, smaller growth yield loss against the ancestor); and there was also an overall effect of higher temperatures to increase relative growth performance (reducing growth yield loss). In addition, the genotype × temperature (*χ*^*2*^_9,522_ = 192.333, *P* < 2 × 10^–16^), carbon × temperature (*χ*^*2*^_5,522_ = 18.214, *P* = 0.003), and genotype × carbon (*χ*^*2*^_45,522_ = 81.950, *P* = 6 × 10^–4^) interaction effects were all significant. The three-way interaction effect, genotype × carbon × temperature, was non-significant (which were removed by model simplification). However, we cannot rule out the possibility that temperature may affect the interaction between genotype and carbon resource, particularly because the statistical model here could not include interaction terms involving a quadratic term of temperature.

**Fig. 3:**
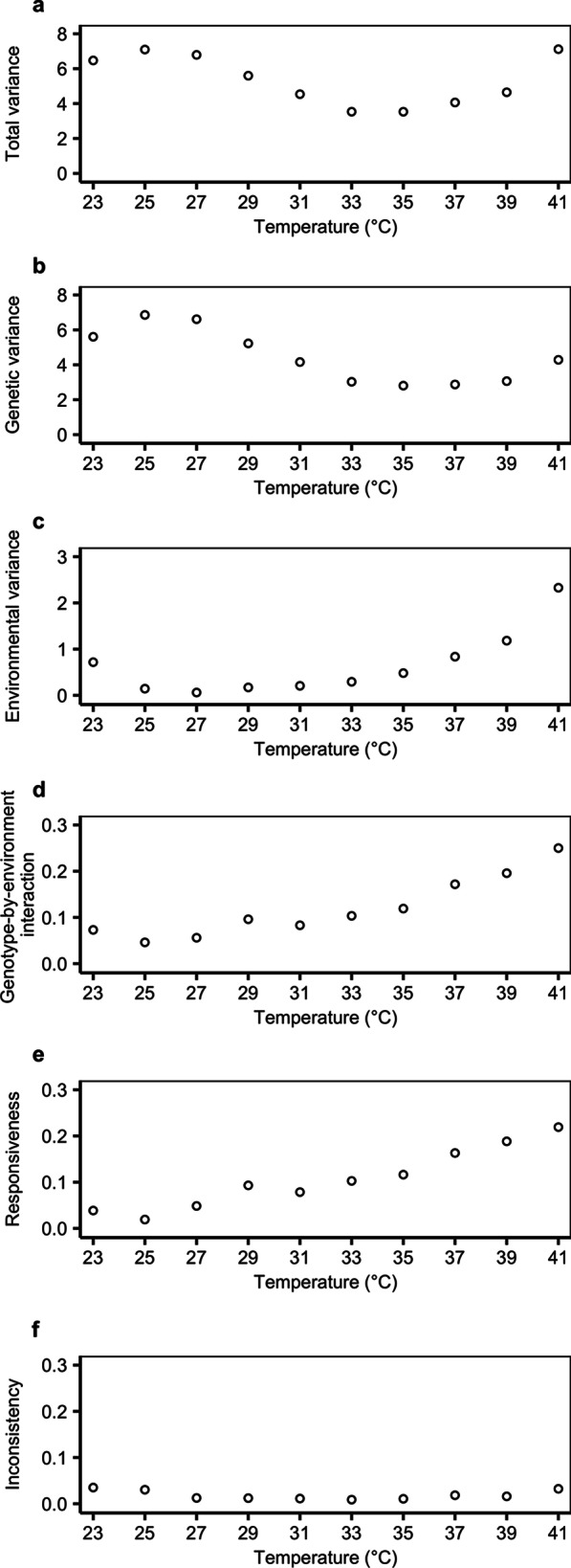
Variation in relative growth performance and its components across different temperatures. **a** Total variance, **b** genetic variance, **c** environmental variance, **d** genotype-by-environment interaction, **e** responsiveness and **f** inconsistency

Variance in relative growth performance at each temperature was partitioned into genetic, environmental and genotype-by-environment interaction components, which was further decomposed into responsiveness and inconsistency as described earlier [40–42]. Total variance was smallest at intermediate temperatures (general linear model, quadratic effect of temperature, *F*_1,7_ = 9.230, *P* = 0.019; linear effect, *F*_1,7_ = 3.216, *P* = 0.116; Fig. 3a; Additional file 1: Table S4). On average, 82% of total variance could be attributable to genetic component, which decreased at higher temperatures (linear effect, *F*_1,8_ = 12.043, *P* = 0.008; quadratic effect non-significant; Fig. 3b; Additional file 1: Table S4). Environmental (carbon resource) variance was smallest at intermediate temperatures and was overall larger at higher temperatures (quadratic effect, *F*_1,7_ = 64.773, *P* = 9 × 10^–5^; linear effect, *F*_1,7_ = 71.308, *P* = 6 × 10^–5^; Fig. 3c; Additional file 1: Table S4). Genotype-by-environment variance was smallest at intermediate (though relatively low) temperatures; and was overall larger at higher temperatures (quadratic effect, *F*_1,7_ = 27.623, *P* = 0.001; linear effect, *F*_1,7_ = 187.729, *P* = 3 × 10^–6^; Fig. 3d; Additional file 1: Table S4). On average, 80% of the genotype-by-environment variance could be attributable to responsiveness. Responsiveness, which arises from the difference in environmental variance among genotypes, increased with temperature (linear effect, *F*_1,7_ = 175.448, *P* = 3 × 10^–6^; quadratic effect marginally significant, *F*_1,7_ = 5.713, *P* = 0.048, with the model estimating an extreme value at a very low temperature that was out of the range of our assay temperatures; Fig. 3e; Additional file 1: Table S4). Inconsistency, which is due to contrasting correlations among genotypes over environments, was smaller at intermediate temperatures (quadratic effect of temperature, *F*_1,7_ = 53.290, *P* = 1 × 10^–4^; linear effect non-significant; Fig. 3f; Additional file 1: Table S4).

**Fig. 4:**
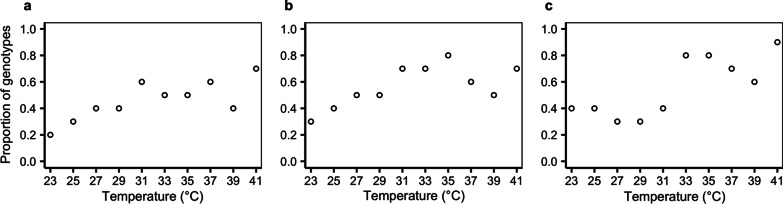
Proportions of genotypes showing resource-dependent growth performance loss across different temperatures. Resource-dependent growth performance loss is defined as showing reduced growth performance on at least one, not all the six, carbon substrates. Criteria for growth performance loss are defines as relative growth < log_10_0.99 (**a**), log_10_0.95 (**b**) or log_10_0.90 (**c**)

We further categorized bacterial genotypes into two types at each temperature: those with ‘resource-independent deleterious’ effects (showing impaired growth performance on all the six carbon substrates) and those with ‘resource-dependent deleterious’ effects (showing reduced performance on at least one, but not all the six, carbon substrates). The proportion of genotypes with resource-dependent deleterious effects increased monotonically with increasing temperatures (*χ*^*2*^_1,8_ = 5.074, *P* = 0.024; *χ*^*2*^_1,8_ = 4.067, *P* = 0.044; *χ*^*2*^_1,8_ = 12.268, *P* = 5 × 10^–5^ for analysis based on growth performance loss definitions as < log_10_0.99, log_10_0.95 and log_10_0.90, respectively; Fig. 4 and Additional file 1: Table S5); and the proportion of genotypes with resource-independent deleterious effects showed a negative relationship with temperature (Additional file 1: Table S5). Including a quadratic term of temperature did not improve the linear models (Additional file 1: Table S5).

## Discussion

The kinetic effects of temperature have important ecological and evolutionary consequences [43–46]. Here we hypothesize that higher temperatures allow for a greater chance of conditional neutrality in fitness effects of MA, based on the universal temperature consequence for speeding rate-limiting physiological processes and thus mitigating the impacts of potentially deleterious mutations (Additional file 1: Fig. S1). This hypothesis is supported by our experiment with MA genotypes of *E. coli*. Those genotypes were very likely to show reduced growth performance on all the six substitutable carbon resources when grown in colder environments and suffer reduced growth performance only on some, not all the six, carbon resources when grown at higher temperatures (Figs. 1, 2). It is known that accumulation of conditionally neutral mutations is a major mechanism underlying fitness trade-offs, and thus local adaptation, among environments [16, 47–49]. If the temperature effects for resource dependence of MA effects observed here are generalizable to other environmental factors (e.g., substitutable nitrogen resources, or compound resources of different qualities), this constitutes an explanation for greater population divergence, and overall greater genetic diversity, in warmer regions [50–52]. It is noteworthy that our prediction may not hold for mutations that impact the stability of proteins (including enzymes) rather than the speed of physiological processes; and fitness effects of those protein stability-related mutations could be ameliorated at relatively lower, not higher, temperatures [37, 53]. Our MA lines may have accumulated very few protein stability-related mutations, and thus supported our hypothesis that is based on temperature effects on rate-limiting physiological processes. It is possible that accumulation of destabilizing mutation can occur under certain conditions; e.g., in populations that have high mutation rates and have evolved for very long time in a constant, isolated, environment [54].

Meanwhile, the overall fitness effects of MA on growth performance was smaller at intermediate temperatures within the temperature range for our *E. coli* strain (Figs. 1, 2), consistent with an earlier finding that mutational effects are more likely to be neutral under benign conditions and more variable in relatively extreme environments [9, 55]. This suggests that populations located in intermediate temperature ranges may be faced with relaxed negative selection and accumulate more spontaneous mutations. More organisms are now faced with hotter climatic conditions; changes in thermal conditions may affect contemporary population adaptation through a number mechanisms including altering population evolutionary potential [56–58]. There is a possibility that changes in mutational load in populations exacerbate the negative effect of temperature elevation on population demography. This is because populations previously located in benign environments may have accumulated many mutations that were conditionally neutral. Those conditionally neutral mutations might show fitness effect if populations are now faced with stressfully hot conditions. However, in case that accumulated mutations become deleterious only in certain, not all, habitats, protection of diverse microhabitats could mitigate the detrimental effect of temperature elevation. Intriguingly, populations located in cold climates that is now becoming warmer may experience relaxed negative selection against MA, consequences of which for population demography and evolutionary adaptation are unknown.

Previous studies of temperature consequences for MA effects have typically been concerned with the impacts of stressfully hot conditions, with a few exceptions that compared two or three temperatures within the ‘normal’ temperature ranges for specific study organisms [10, 12, 59–61]. Higher temperatures were found to mitigate fitness effects of MA in some, not all, studies [10]; and the temperature consequences for genotype-by-environment interactions has been poorly understood. Obviously more studies covering a wider range of temperatures representative of study organisms’ fundamental thermal niche space would be helpful for a more comprehensive understanding of MA effects.

## Conclusions

Mutation accumulation is more likely to show resource-dependent neutral fitness effects at higher, relative to lower, temperatures. This is a possible mechanism to cause greater population divergence in warmer areas, and thus an overall pattern of increasing genetic diversity with decreasing latitudes.

## Methods

### The mutation accumulation (MA) experiment

MA experiments have been widely used for studying mutational effects. Evolution lines in MA experiments typically have very small effective population sizes; thus selection is minimized and spontaneous mutations, except for lethal ones, could get fixation through drift [10, 62, 63]. The present study used ten *Escherichia coli* genotypes from an earlier MA experiment [64]*.* All the ten genotypes went through 30 bottlenecks at 37 °C (approximately 790 generations). For each MA line, one bottleneck of evolution involved randomly choosing a single colony and streaking onto a new LB-Miller agar plate for a new round of 24 h growth. The number of mutations (base-pair substitutions) in the ten MA genotypes ranged between 63 and 101 (Additional file 1: Table S6). No signal of selection during MA was found for the genotypes used in the present study [64].

### Growth performance measurement

Growth yields were used to estimate growth performance as they reflect the ability of genotypes to acquire and convert nutrients into total biomass [65]. Specifically, growth yields of the ten MA genotypes and the ancestral strain were measured in six liquid media at ten temperatures by measuring the optical density (OD). The six liquid media differed in carbon substrates; they consisted of Davis minimal medium [66] supplemented with one of the following carbon resources at a concentration of 0.4 g L^−1^: fructose (13.3 mM of carbon atoms), galactose (13.3 mM of C), glucose (13.3 mM of C), glycerol (13.0 mM of C), maltose (14.0 mM of C) and trehalose (14.0 mM of C). The six carbon sources cover the four categories of transport mechanisms across outer and inner membranes [67]. Glucose and fructose belong to the OmpF/PTS type that pass through the outer membrane via the porin OmpF and then cross inner membrane via the phosphotransferase system (PTS). Galactose and glycerol are of the OmpF/non-PTS type that pass through the outer membrane via OmpF and inner membrane via other nutrient-specific proteins that are not part of PTS. Trehalose is a LamB/PTS nutrient that passes through the outer and inner membrane primarily via the larger diameter porin LamB and PTS, respectively. Maltose is a LamB/non-PTS nutrient. All the six carbon substrates could well support the growth of ancestral strain at 37 °C (Additional file 1: Fig. S2). The ten assay temperatures, 23, 25, 27, 29, 31, 33, 35, 37, 39 and 41 °C, covered the normal thermal range for our study bacterial strain whose lower and upper temperature limits were ~ 19 and ~ 42.2 °C respectively where the bacterium fails to maintain a viable population in face of serial transfer of 1:100 dilution per day [68, 69]. Ten incubators were used for the assays, one for each temperature. Each assay was repeated three times and mean values of these technical replicates were used in data analysis.

Bacteria were grown in 4 mL of nutrient media (in 50 mL centrifuge tubes with loosen caps) with shaking frequency ~ 400 rpm. Frozen stocked samples were reconditioned in LB-Miller broth at 37 °C for 24 h, followed by 24 h acclimation in each assay environment (with 0.4 μL of the reconditioned culture as inoculum). Then 40 μL of each acclimated culture was transferred to fresh medium for a new round of 24 h incubation during which all cultures could reach their stationary phases. Growth yield (i.e. the carrying capacity) of each culture was estimated by measuring the optical density (OD) [24]. Each culture was vortexed, 200 μL of which was loaded into a well of the 96-well microplate, milli-OD scores (mOD) at 600 nm were measured using a microplate reader (PowerWave XS2, Bio-Tek Instruments, Inc., Winooski, VT, USA). A ‘blank’ well that contained 200 μL of fresh medium was also measured, giving a background mOD value. The background mOD value was subtracted from the measured mOD values of each culture. A relative growth performance score was calculated for each MA genotype in each assay environment as ${log}_{10}\frac{{mOD}_{MA} +1 }{{mOD}_{ancestor}+1}$, where 1 was added to give valid values in case OD_600_ was read as zero. A positive score indicates an increase in growth performance compared with the ancestral type and a negative value suggests a decrease.

### Statistical analysis

Data analysis was carried out using R 3.5.2 [70]. All statistical models used here were subject to model simplification that stepwise removed non-significant effects [71]. First, we examined how relative growth performance changed with assay temperatures, using a linear mixed-effect model. In this model, genotype and carbon substrate were included as categorical explanatory variables, temperature was a continuous explanatory variable, and a quadratic term of temperature was also included (which was not involved in any interaction terms); and incubator ID was included as a random factor. The ‘Anova’ function provided by the ‘car’ package was used to estimate the statistical significance of the effect of each explanatory variable.

Second, we investigated how temperature may alter resource dependence of relative growth performance. Fitness rank curves were drawn to visualize the dependence of relative growth performance on genotype and carbon resource at each temperature, as [24, 72, 73]. Quantitative analysis was carried out based on variance partitioning. Variance in relative growth performance of MA genotypes at each temperature was partitioned into genetic (${{\sigma }^{2}}_{G}$), environmental (mainly caused by different carbon substrate; ${{\sigma }^{2}}_{E}$), and genotype-by-environment interaction (${{\sigma }^{2}}_{GE}$) components. The interaction term was further decomposed into two parts, responsiveness (*R*) and inconsistency (*I*): ${{\sigma }^{2}}_{GE}= R+I$. Responsiveness is calculated as $R= \sum \frac{{\left({\sigma }_{Ei}- {\sigma }_{Ej}\right)}^{2}}{2G(G-1)}$, where ${\sigma }_{Ei}$ and ${\sigma }_{Ej}$ are the environmental standard deviations of relative growth performance scores expressed by genotypes *i* and *j*, respectively, and *G* is the number of genotypes tested. The inconsistency is calculated as $I= \sum \frac{{\sigma }_{Ei}{\sigma }_{Ej}\left(1-{\sigma }_{EiEj}\right)}{G(G-1)}$, where ${\sigma }_{EiEj}$ is the environmental correlation of relative growth performance values across the two genotypes. The relationship between each of the variance components and temperature, the relationship between the proportion of resource-dependent deleterious MA genotypes (out of a total of 10) and temperature, and the relationship between the proportion of resource-independent deleterious MA genotypes and temperature were analyzed using the general linear model where a linear term and a quadratic term of temperature were included. The ‘Anova’ function provided by the ‘car’ package was used to estimate the significance of effects of the explanatory variables. Separate analyses were performed based on three criteria to define growth performance loss, that is, relative growth performance < log_10_0.99, log_10_0.95, or log_10_0.90.

## Supplementary Information

**Additional file 1: Table S1.** Mean relative growth performance of MA genotypes in each assay environment. **Table S2.** Correlation between growth performance scores and the number of base-pair substitutions (BPSs) occurred in coding regions in each assay environment. **Table S3.** Correlation between growth performances and the number of base-pair substitutions (BPSs) in carbohydrate metabolism related genes of MA lines in each assay environment. **Table S4.** Summary of statistical models for the temperature response of growth performance variance components. **Table S5.** Summary of statistical models for the temperature response of the frequency of two categories of genotypes. **Table S6.** Number of base-pair substitutions (BPSs) accumulated in each MA genotype. **Figure S1.** An illustration of temperature-dependent mutation accumulation (MA) effects on hypothetical fitness landscapes. **Figure S2**. Growth performance of the ancestral strain across different assay environments.

## Data Availability

Data associated with this study are available at figshare (https://doi.org/10.6084/m9.figshare.12473036).
