# Supplementary material for: Consequences of mutation accumulation for growth performance are more likely to be resource-dependent at higher temperatures
Source: BMC Ecol Evol. 2021 Jun 6;21:109. doi: 10.1186/s12862-021-01846-1 (PMC8180013; doi:10.1186/s12862-021-01846-1)

**Additional material**

**Table S1.** Mean relative growth performance of MA genotypes in each assay environment (mean ± sd, n = 10).

| Temperature (°C) | Carbon substrate | | | | | |
| --- | --- | --- | --- | --- | --- | --- |
|  | Fructose | Galactose | Glucose | Glycerol | Maltose | Trehalose |
| 23 | -0.481 ± 0.373 | -1.072 ± 0.183 | -0.825 ± 0.279 | -0.520 ± 0.255 | -1.018 ± 0.268 | -1.118 ± 0.234 |
| 25 | -0.608 ± 0.405 | -0.844 ± 0.151 | -0.740 ± 0.220 | -0.955 ± 0.104 | -0.871 ± 0.172 | -0.898 ± 0.146 |
| 27 | -0.810 ± 0.217 | -0.775 ± 0.136 | -0.620 ± 0.462 | -0.867 ± 0.251 | -0.759 ± 0.150 | -0.760 ± 0.048 |
| 29 | -0.720 ± 0.318 | -0.603 ± 0.105 | -0.451 ± 0.412 | -0.833 ± 0.433 | -0.604 ± 0.302 | -0.522 ± 0.266 |
| 31 | -0.600 ± 0.196 | -0.483 ± 0.178 | -0.337 ± 0.361 | -0.718 ± 0.408 | -0.473 ± 0.169 | -0.330 ± 0.379 |
| 33 | -0.491 ± 0.371 | -0.311 ± 0.151 | -0.173 ± 0.401 | -0.669 ± 0.445 | -0.324 ± 0.167 | -0.256 ± 0.369 |
| 35 | -0.341 ± 0.204 | -0.273 ± 0.173 | -0.215 ± 0.408 | -0.818 ± 0.690 | -0.284 ± 0.224 | -0.218 ± 0.268 |
| 37 | -0.452 ± 0.226 | -0.464 ± 0.401 | -0.174 ± 0.401 | -1.036 ± 0.713 | -0.299 ± 0.324 | -0.317 ± 0.319 |
| 39 | -0.636 ± 0.404 | -0.693 ± 0.408 | -0.230 ± 0.373 | -1.216 ± 0.683 | -0.340 ± 0.321 | -0.354 ± 0.432 |
| 41 | -1.165 ± 0.349 | -1.344 ± 0.447 | -0.806 ± 0.521 | 0.090 ± 0.695 | -1.061 ± 0.214 | -0.769 ± 0.622 |

**Table S2.** Correlation between growth performance scores and the number of base-pair substitutions (BPSs) occurred in coding regions in each assay environment (Pearson's correlation test, df = 8 for each test).

|  |  | Fructose | Galactose | Glucose | Glycerol | Maltose | Trehalose |
| --- | --- | --- | --- | --- | --- | --- | --- |
| 23°C | *r* | -0.198 | -0.097 | -0.145 | -0.179 | -0.135 | -0.187 |
|  | *P* | 0.584 | 0.789 | 0.690 | 0.622 | 0.709 | 0.605 |
| 25°C | *r* | -0.074 | -0.140 | -0.165 | -0.113 | -0.111 | -0.140 |
|  | *P* | 0.839 | 0.699 | 0.648 | 0.755 | 0.760 | 0.700 |
| 27°C | *r* | -0.083 | -0.152 | -0.256 | -0.102 | -0.221 | -0.179 |
|  | *P* | 0.820 | 0.674 | 0.476 | 0.778 | 0.540 | 0.620 |
| 29°C | *r* | -0.180 | -0.207 | -0.216 | -0.126 | -0.315 | -0.270 |
|  | *P* | 0.618 | 0.566 | 0.550 | 0.729 | 0.376 | 0.451 |
| 31°C | *r* | -0.249 | -0.267 | -0.258 | -0.175 | -0.291 | -0.133 |
|  | *P* | 0.489 | 0.456 | 0.472 | 0.629 | 0.415 | 0.715 |
| 33°C | *r* | -0.257 | -0.180 | 0.022 | -0.211 | -0.084 | 0.009 |
|  | *P* | 0.473 | 0.618 | 0.951 | 0.558 | 0.818 | 0.981 |
| 35°C | *r* | -0.149 | -0.088 | 0.074 | -0.139 | 0.035 | 0.087 |
|  | *P* | 0.681 | 0.808 | 0.839 | 0.702 | 0.923 | 0.811 |
| 37°C | *r* | -0.049 | -0.047 | 0.319 | 0.104 | 0.212 | 0.283 |
|  | *P* | 0.893 | 0.898 | 0.368 | 0.775 | 0.556 | 0.429 |
| 39°C | *r* | -0.057 | 0.041 | 0.401 | -0.103 | 0.279 | 0.305 |
|  | *P* | 0.876 | 0.910 | 0.250 | 0.777 | 0.434 | 0.391 |
| 41°C | *r* | 0.141 | 0.292 | -0.030 | -0.426 | 0.190 | 0.358 |
|  | *P* | 0.697 | 0.413 | 0.934 | 0.219 | 0.600 | 0.310 |

**Table S3.** Correlation between growth performances and the number of base-pair substitutions (BPSs) in carbohydrate metabolism related genes of MA lines in each assay environment (Pearson's correlation test, df = 8 for each test).

|  |  | Fructose | Galactose | Glucose | Glycerol | Maltose | Trehalose |
| --- | --- | --- | --- | --- | --- | --- | --- |
| 23°C | *r* | -0.556 | -0.339 | -0.250 | -0.429 | -0.260 | -0.360 |
|  | *P* | 0.095 | 0.338 | 0.487 | 0.216 | 0.469 | 0.307 |
| 25°C | *r* | -0.380 | -0.180 | -0.184 | -0.264 | -0.153 | -0.215 |
|  | *P* | 0.278 | 0.619 | 0.612 | 0.462 | 0.672 | 0.551 |
| 27°C | *r* | -0.177 | -0.183 | -0.130 | -0.171 | -0.178 | -0.182 |
|  | *P* | 0.625 | 0.612 | 0.720 | 0.636 | 0.623 | 0.616 |
| 29°C | *r* | -0.189 | -0.154 | -0.057 | -0.177 | -0.151 | -0.163 |
|  | *P* | 0.602 | 0.670 | 0.875 | 0.626 | 0.678 | 0.653 |
| 31°C | *r* | -0.130 | -0.108 | -0.250 | -0.176 | -0.093 | 0.136 |
|  | *P* | 0.721 | 0.766 | 0.486 | 0.628 | 0.798 | 0.709 |
| 33°C | *r* | -0.085 | 0.083 | 0.312 | -0.135 | 0.223 | 0.361 |
|  | *P* | 0.815 | 0.820 | 0.380 | 0.710 | 0.535 | 0.306 |
| 35°C | *r* | 0.064 | 0.211 | 0.383 | -0.193 | 0.330 | 0.365 |
|  | *P* | 0.860 | 0.558 | 0.275 | 0.592 | 0.351 | 0.300 |
| 37°C | *r* | 0.093 | 0.001 | 0.329 | -0.304 | 0.361 | 0.333 |
|  | *P* | 0.799 | 0.999 | 0.353 | 0.394 | 0.305 | 0.348 |
| 39°C | *r* | -0.092 | -0.173 | 0.280 | -0.452 | 0.299 | 0.333 |
|  | *P* | 0.801 | 0.633 | 0.433 | 0.189 | 0.401 | 0.347 |
| 41°C | *r* | 0.290 | 0.184 | -0.098 | -0.181 | 0.238 | 0.471 |
|  | *P* | 0.416 | 0.611 | 0.789 | 0.617 | 0.508 | 0.170 |

**Table S4.** Summary of statistical models for the temperature response of growth performance variance components. Shown here are models with both a linear term and a quadratic term of temperature, and if appropriate, simplified models with only a linear term. ${\sigma^{2}}_{G}$, genetic variance; ${\sigma^{2}}_{E}$, environmental variance; ${\sigma^{2}}_{GE}$, genotype-by-environment interaction; *R*, responsiveness; *I*, inconsistency. Estimated temperatures are those correspond to the extreme values of response variables; and ‘NA’ indicates where the quadratic effect of temperature was non-significant and temperature corresponding to extreme value was not estimated.

|  | Model with linear and quadratic terms | | | | |  | Simplified model with only a linear term | | | |
| --- | --- | --- | --- | --- | --- | --- | --- | --- | --- | --- |
|  | Effect | df | *F* | *P* | Estimated temperature (°C) |  | Effect | df | *F* | *P* |
| Total variance | Linear | 1 | 3.216 | 0.116 | 33.495 |  |  |  |  |  |
|  | Quadratic | 1 | 9.230 | 0.019 |  |  |  |  |  |  |
|  | Error | 7 |  |  |  |  |  |  |  |  |
| ${\sigma^{2}}_{G}$ | Linear | 1 | 14.041 | 0.007 | NA |  | Linear | 1 | 12.043 | 0.008 |
|  | Quadratic | 1 | 2.327 | 0.171 |  |  | Error | 8 |  |  |
|  | Error | 7 |  |  |  |  |  |  |  |  |
| ${\sigma^{2}}_{E}$ | Linear | 1 | 71.308 | 6E-05 | 29.346 |  |  |  |  |  |
|  | Quadratic | 1 | 64.773 | 9E-05 |  |  |  |  |  |  |
|  | Error | 7 |  |  |  |  |  |  |  |  |
| ${\sigma^{2}}_{GE}$ | Linear | 1 | 187.729 | 3E-06 | 25.406 |  |  |  |  |  |
|  | Quadratic | 1 | 27.623 | 0.001 |  |  |  |  |  |  |
|  | Error | 7 |  |  |  |  |  |  |  |  |
| *R* | Linear | 1 | 175.448 | 3E-06 | 17.978 |  |  |  |  |  |
|  | Quadratic | 1 | 5.713 | 0.048 |  |  |  |  |  |  |
|  | Error | 7 |  |  |  |  |  |  |  |  |
| *I* | Linear | 1 | 2.224 | 0.179 | 32.528 |  |  |  |  |  |
|  | Quadratic | 1 | 53.290 | 1E-04 |  |  |  |  |  |  |
|  | Error | 7 |  |  |  |  |  |  |  |  |

**Table S5.** Summary of statistical models for the temperature response of the frequencies of two categories of genotypes. Shown here are models with both a linear and a quadratic term of temperature, and if appropriate, simplified models with only a linear term. Genotypes with resource-dependent growth performance loss refer to those showing impaired growth performance on at least one, but not all the six, substrates; and genotypes with resource-independent growth performance loss are those showing growth performance loss on all the six substrates.

|  | Growth performance loss defined as relative growth < | Model with linear and quadratic terms | | | |  | Simplified model with only a linear term | | | |
| --- | --- | --- | --- | --- | --- | --- | --- | --- | --- | --- |
|  |  | Effect | df | *χ^2^* | P |  | Effect | df | *χ^2^* | P |
| Resource-dependent growth performance loss | log_10_0.99 | Linear | 1 | 0.918 | 0.338 |  | Linear | 1 | 5.074 | 0.024 |
|  |  | Quadratic | 1 | 0.619 | 0.432 |  | Error | 8 |  |  |
|  |  | Error | 7 |  |  |  |  |  |  |  |
|  | log_10_0.95 | Linear | 1 | 2.910 | 0.088 |  | Linear | 1 | 4.067 | 0.044 |
|  |  | Quadratic | 1 | 2.413 | 0.120 |  | Error | 8 |  |  |
|  |  | Error | 7 |  |  |  |  |  |  |  |
|  | log_10_0.90 | Linear | 1 | 0.098 | 0.755 |  | Linear | 1 | 12.268 | 5E-05 |
|  |  | Quadratic | 1 | 0.336 | 0.562 |  | Error | 8 |  |  |
|  |  | Error | 7 |  |  |  |  |  |  |  |
| Resource-independent growth performance loss | log_10_0.99 | Linear | 1 | 0.918 | 0.338 |  | Linear | 1 | 5.074 | 0.024 |
|  |  | Quadratic | 1 | 0.619 | 0.432 |  | Error | 8 |  |  |
|  |  | Error | 7 |  |  |  |  |  |  |  |
|  | log_10_0.95 | Linear | 1 | 2.472 | 0.116 |  | Linear | 1 | 3.14 | 0.076 |
|  |  | Quadratic | 1 | 2.067 | 0.151 |  | Error | 8 |  |  |
|  |  | Error | 7 |  |  |  |  |  |  |  |
|  | log_10_0.90 | Linear | 1 | 0.827 | 0.363 |  | Linear | 1 | 7.277 | 0.007 |
|  |  | Quadratic | 1 | 0.492 | 0.483 |  | Error | 8 |  |  |
|  |  | Error | 7 |  |  |  |  |  |  |  |

**Table S6.** Number of base pair substitutions (BPSs) accumulated in each MA genotype. The accumulated BPS mutations were classified as non-coding and coding BPSs based on their consequences; BPSs occurred in coding regions were further counted according to the annotated metabolic pathways. Metabolic pathways of the ancestral strain were constructed based on the KEGG database at Novogene.

| ID | BPS | | | |
| --- | --- | --- | --- | --- |
|  | Total | Non-coding | Coding | Carbohydrate  metabolism related |
| A1 | 101 | 9 | 92 | 8 |
| A2 | 93 | 11 | 82 | 8 |
| A3 | 86 | 11 | 75 | 4 |
| A4 | 84 | 5 | 79 | 7 |
| A5 | 78 | 11 | 67 | 2 |
| A6 | 74 | 8 | 66 | 5 |
| A7 | 63 | 6 | 57 | 7 |
| A8 | 72 | 6 | 66 | 7 |
| A9 | 98 | 14 | 84 | 10 |
| A10 | 92 | 8 | 84 | 10 |

**Figure S1.** An illustration of temperature-dependent mutation accumulation (MA) effects on hypothetical fitness landscapes. Mutation accumulation in the absence of selection is akin to a random walk away from the wild-type genotype (white lines and arrows). In cold environments, mutation accumulation may cause similar fitness-loss effects regardless of environmental conditions (**A** and **B**). Warmer temperatures may mitigate the fitness consequences of some mutations, particularly those associated with rate-limiting physiological processes; therefore, MA more likely to cause (nearly) neutral fitness effects or smaller fitness loss in warmer environments. When the mitigation effects of high temperatures for mutational consequences vary among habitats (**C** and **D**), environmental dependence in MA effects, including the chance of conditional neutrality, would also be greater.


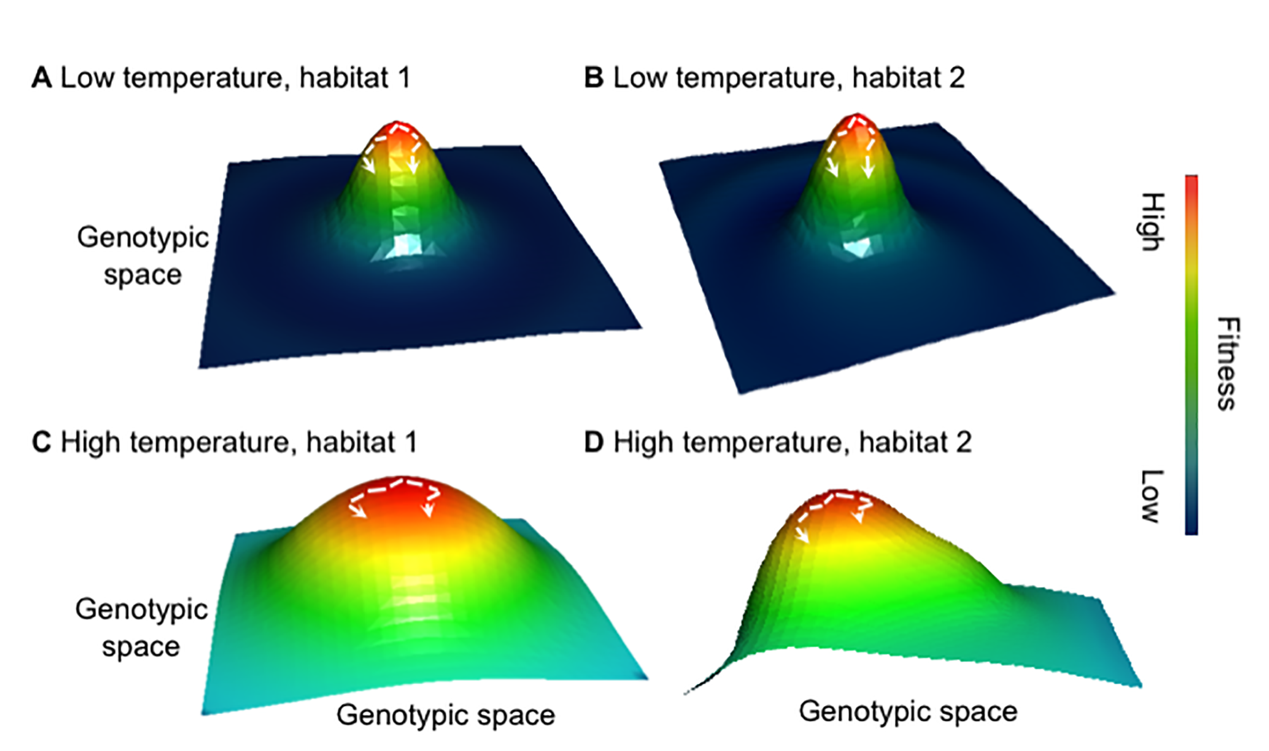


**Figure S2.** Growth performance of the ancestral strain across different assay environments. Growth performance was shown as log_10_(mOD_600_+1).


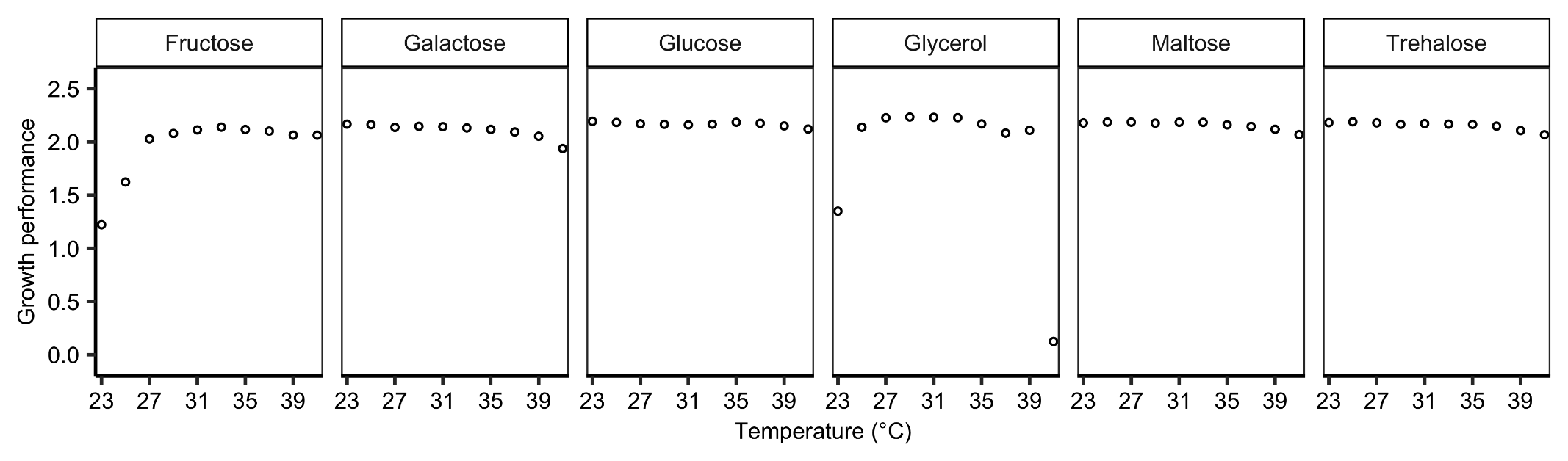

Supplement: Supplementary file 1 — Additional file 1: Table S1. Mean relative growth performance of MA genotypes in each assay environment. Table S2. Correlation between growth performance scores and the number of base-pair substitutions (BPSs) occurred in coding regions in each assay environment. Table S3. Correlation between growth performances and the number of base-pair substitutions (BPSs) in carbohydrate metabolism related genes of MA lines in each assay environment. Table S4. Summary of statistical models for the temperature response of growth performance variance components. Table S5. Summary of statistical models for the temperature response of the frequency of two categories of genotypes. Table S6. Number of base-pair substitutions (BPSs) accumulated in each MA genotype. Figure S1. An illustration of temperature-dependent mutation accumulation (MA) effects on hypothetical fitness landscapes. Figure S2. Growth performance of the ancestral strain across different assay environments. [file 12862_2021_1846_MOESM1_ESM.docx]
